# Long-term clinical outcome of tendon transfer and tendon graft for extensor tendon ruptures in rheumatoid hands

**DOI:** 10.1186/s12891-022-05815-7

**Published:** 2022-09-16

**Authors:** Young Seok Lee, Hee Soo Kim, Yeong Hwan Kim, Young-Hoon Jo, Bong Gun Lee, Chang-Hun Lee

**Affiliations:** 1 Department of Orthopedic Surgery, Hanyang University Guri Hospital, Guri, Republic of Korea; 2 Department of Orthopedic Surgery, Saeum Hospital, Seoul, Republic of Korea; 3 Department of Orthopedic Surgery, College of Medicine, Hanyang University, Seoul, Republic of Korea

**Keywords:** Tendon, Transfer, Graft, Rheumatoid arthritis, Rupture

## Abstract

**Purpose:**

To evaluate the objective and subjective long-term clinical outcomes of tendon transfer and tendon graft for extensor tendon ruptures in rheumatoid hands.

**Methods:**

We evaluated the long-term clinical outcomes of tendon transfer and tendon graft for extensor tendon ruptures in rheumatoid hands of 37 patients (43 hands) followed up for a mean of 14 years (range, 10–21 years).

**Results:**

The mean time from rupture to surgery was 13.1 weeks (range, 3–48 weeks). The mean extension lag of the metacarpophalangeal joint was 8.7° (range, 0–40°), the mean pulp-to-palm distance was 0.4 cm (range, 0–3 cm), and the mean overall satisfaction rate was 86.5 (range, 70–100). There were no significant differences in clinical outcomes between tendon transfers and tendon grafts. There was a significant correlation between extension lag of the metacarpophalangeal joint and overall satisfaction rate (*R*^2^ = 0.155; *p* = 0.009). Time to surgery was significantly correlated with extension lag of the metacarpophalangeal joint (*R*^2^ = 0.437; *p* = 0.001) in the tendon graft group.

**Conclusions:**

Both tendon transfer and tendon graft for extensor tendon ruptures in rheumatoid hands achieve satisfactory results that are maintained for an average of 14 years. In cases of tendon graft, the time to surgery should be considered, and there is concern over extension lag of MP joint.

**Level of Evidence:**

IV

## Introduction

Rheumatoid arthritis (RA) is a chronic, progressive autoimmune disease that affects the hand in almost 90% of patients [1]. Spontaneous extensor tendon rupture, which is frequently seen in rheumatoid hands, is associated with several risk factors such as persistent synovitis, scallop sign on radiography, and dorsal subluxation of the distal ulnar head, also called caput ulnae syndrome [2]. Although joint destruction may be a major cause of functional impairment, extensor tendon rupture, if not properly treated at an early stage, can lead to severe hand dysfunction.

Tendon transfer and tendon grafting are the preferred methods over end-to-end primary repair for RA extensor tendon rupture because, in the latter, flexion is limited after resection of a poor stump [3]. Tendon transfer is the primary treatment for restoring extensor function, and many authors have reported satisfactory results with such treatments [4, 5]. Despite concerns about adherence to the inflammatory bed and muscle contracture [3, 6], Bora et al. reported favorable results in 23 patients who underwent free tendon grafts for extensor tendon rupture in rheumatoid hands. In addition, Mountney et al. documented that the interposition palmaris longus (PL) tendon graft implements an anatomical axis and provides greater forces in the biomechanical model [7, 8].

Satisfactory results with both tendon transfer and tendon graft in similar patient groups have been reported, with a mean follow-up time of 5.6 years [9]. However, most previous studies are only on the short- or medium-term follow-up results. Thus, this study aimed to evaluate the objective and subjective long-term clinical outcomes of tendon transfer and tendon grafting for extensor tendon ruptures in rheumatoid hands.

## Methods

### Study design and subjects

We retrospectively reviewed patients who had undergone tendon transfer or tendon grafting due to spontaneous tendon rupture in the rheumatoid hands at our orthopedic department. From an initial pool of 62 patients who began treatment between August 1995 and August 2007, 51 patients followed up for at least 10 years were evaluated. Among them, five patients with rupture of the extensor pollicis longus and six patients with rupture of the flexor tendon were excluded. After further excluding one patient hospitalized at another hospital due to other illnesses, two patients who died, and three patients who refused follow-up because of their distance from the hospital, 37 patients (43 hands) were finally included in the study.

Among them, 5 and 38 were men and women, respectively. The mean age at the time of surgery was 47 years (range, 26–62 years). There were 29 and 14 cases of right and left hand involvement, respectively. The mean duration of RA was 11.4 years (range 2–18), and all patients were receiving medication at our rheumatology center. Preoperative radiographs were used to classify the degree of joint destruction according to the Larsen-Dale-Eek classification [10]. There were 1, 15, 13, and 14 cases of grade II, III, IV, and V destruction, respectively.

All methods used in this longitudinal observational study were carried out in accordance with ‘Declaration of Helsinki’, and the study protocol was approved by our institutional review board and we obtained informed consent from the patient(HYUH-2018–01-018).

### Surgical technique

Surgeries were performed in the supine position under general anesthesia. The tourniquet was inflated to a pressure of 250 mmHg on the upper arm, and a curvilinear incision was made in the dorsum of the wrist. The extensor retinaculum was exposed by retracting the flap containing the subcutaneous fat layer to minimize blood vessel and nerve damage. This was followed by an incision in the fourth compartment of the retinaculum, exposing the extensor tendons for synovectomy. The terminal branch was removed by exposing the posterior interosseous nerve, which was located on the floor of the fourth compartment. An incision was made on the septum of the extensor retinaculum for synovectomy of the required compartments. In many cases, the wrist joint capsule, which is located deep into the extensor tendon, was ruptured, and thus, an additional incision was made to remove the hypertrophic synovium of the wrist and distal radioulnar joints.

All bone spurs were removed using a rongeur to prevent re-rupture and, if needed, an oscillating saw was used for hemiresection of the ulnar head, leaving only the styloid process. Hemiresection was performed on 39 cases and wrist joint synovectomy without additional ulna procedures was performed on 4 cases. Out of the total 43 cases, Sauve-Kapandji procedure or Darrach procedure was not performed.

Relocation was performed in cases of subluxation of the extensor carpi ulnaris. Problematic tissue in the proximal and distal stumps of the ruptured extensor tendon was removed, followed by either a tendon transfer or tendon graft, subject to the surgeon’s assessment. For tendon transfers, either an end-to-side suture or an extensor indicis propius (EIP) was employed. Out of the 17 cases of tendon transfer, 12 cases were EIP transfer to fourth EDC and EDM (In cases of fourth, fifth EDC rupture), 3 cases were EIP transfer to fourth EDC and EDM with end-to-side repair of second, third EDC (In cases of third, fourth, fifth EDC rupture). 2 cases were EIP transfer to fifth EDC and EDM. (In cases of fifth EDC, EDM rupture). For tendon grafts, PLs were harvested.

The tendon sutures were performed using the Pulvertaft weave technique to obtain sufficient force. To avoid postoperative loss of tension, the suture was performed in a state in which the wrist was flexed and the fingers were slightly more extended than the tension of the normal tension effect to ensure that grip was possible afterwards. The extensor retinaculum was placed below the tendon to provide a soft bed. The patients wore splints for 3 weeks postoperatively with their fingers and wrists in a fully extended state. Rehabilitation exercises began 3 weeks after the splint was removed.

### Clinical evaluation

Clinical evaluations of all patients were performed by two orthopedic surgeons. A goniometer was used as an objective measure to evaluate the extension lag and range of motion of the metacarpophalangeal, proximal interphalangeal, and distal interphalangeal joints. Pulp-to-palm distance was measured using a ruler, and the measurements were then scored according to Geldmacher criteria [11]. For subjective indicators, the patient was asked to score their satisfaction from 0 to 100, with 0 indicating complete dissatisfaction and 100 indicating complete satisfaction. We also assessed feasibility of performing daily activities (Y/N) and the need to change jobs following the surgery (Y/N).

### Statistical analysis

For statistical analysis, SPSS ver. 27.0 (SPSS Inc., Chicago, IL, USA) was used, and a *p* value < 0.05 was considered statistically significant. For comparisons between the tendon transfer and tendon graft groups, a Student’s t-test was used for parametric variables for a normality test, while the Mann–Whitney test or Kruskal–Wallis test was used for nonparametric variables. Simple linear regression analyses were used to identify demographic data associated with clinical outcomes and outcome variables that affect the overall satisfaction rate.

## Results

**Fig. 1:**
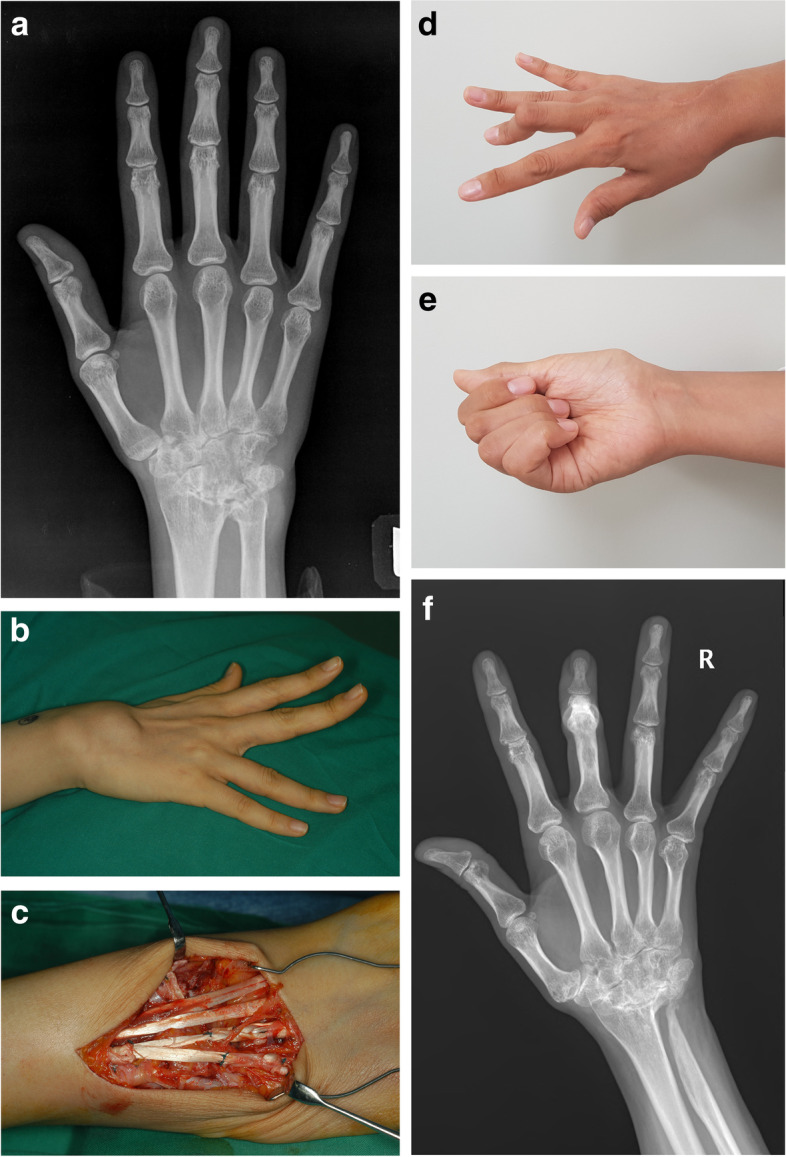
A 36-year-old female patient working as a casino dealer who underwent surgery 124 months ago. **a** Preoperative wrist PA view. **b** Preoperative photograph showing extension lag on the fourth and fifth fingers. **c** Intraoperative photograph showing a tendon graft using PL. **d**, **e** Photographs taken at last outpatient visit. **f** Wrist PA view taken at last outpatient visit. The fourth and fifth fingers were maintained well without extension lag, but the third finger had buttonhole deformity due to the progression of rheumatoid arthritis

The mean follow-up period was 14 years (range, 10–21 years), and the average time from rupture to surgery was 13.1 weeks (range, 3–48 weaks). There were 7, 27, and 9 cases of 1-, 2-, and 3-digit involvement, respectively. In total, 42 patients had a rupture of the extensor digiti minimi or the fifth extensor digitorum communis, 36 patients that had a rupture of the fourth extensor digitorum communis, and 10 patients had a rupture of the third extensor digitorum communis(Fig. 1).

In 3 patients with 6-digit involvement, one patient had dislocation or deformity of the metacarpophalangeal joint due to RA, and the tendon was visible but impossible to functionally assess. Re-rupture occurred in three cases with 7-digit involvement, all subsequent to a tendon graft. Re-rupture occurred in the third, fourth, and fifth fingers in 2 cases, and in only in the fifth finger and not in the reconstructed fourth finger in one patient. The former two patients underwent a second tendon transfer surgery in a different hospital; in the latter, where only the fifth digit was ruptured, the patient decided to forego any further surgeries.

Excluding the three patients of deformity and the three patients of re-rupture, the 74 digits had a mean extension lag of 8.7°(range, 0–40°), a mean range of motion of 75.5° (range, 20–90°), a mean pulp-to-palm distance of 0.4 cm (range, 0–3 cm), and a mean Geldmacher score of 20.7 (range, 8–24). The overall satisfaction rate was 86.5 (range, 70–100) on average (Table 1).

**Table 1:** Outcomes of extensor tendon reconstruction in rheumatoid hands within a mean follow-up of 14 years

Outcome	Value
Deformity of MP joint	6 fingers of 3 cases
Rerupture	7 fingers of 3 cases
Extension lag of MP joint (°)	8.7 (0–40)
Range of motion of MP joint(°)	75.5 (20–90)
Pulp-to-palm distance (cm)	0.4 (0–3)
Geldmacher score	20.7 (8–24)
Overall satisfaction rate	86.5 (0–100)

**Fig. 2:**
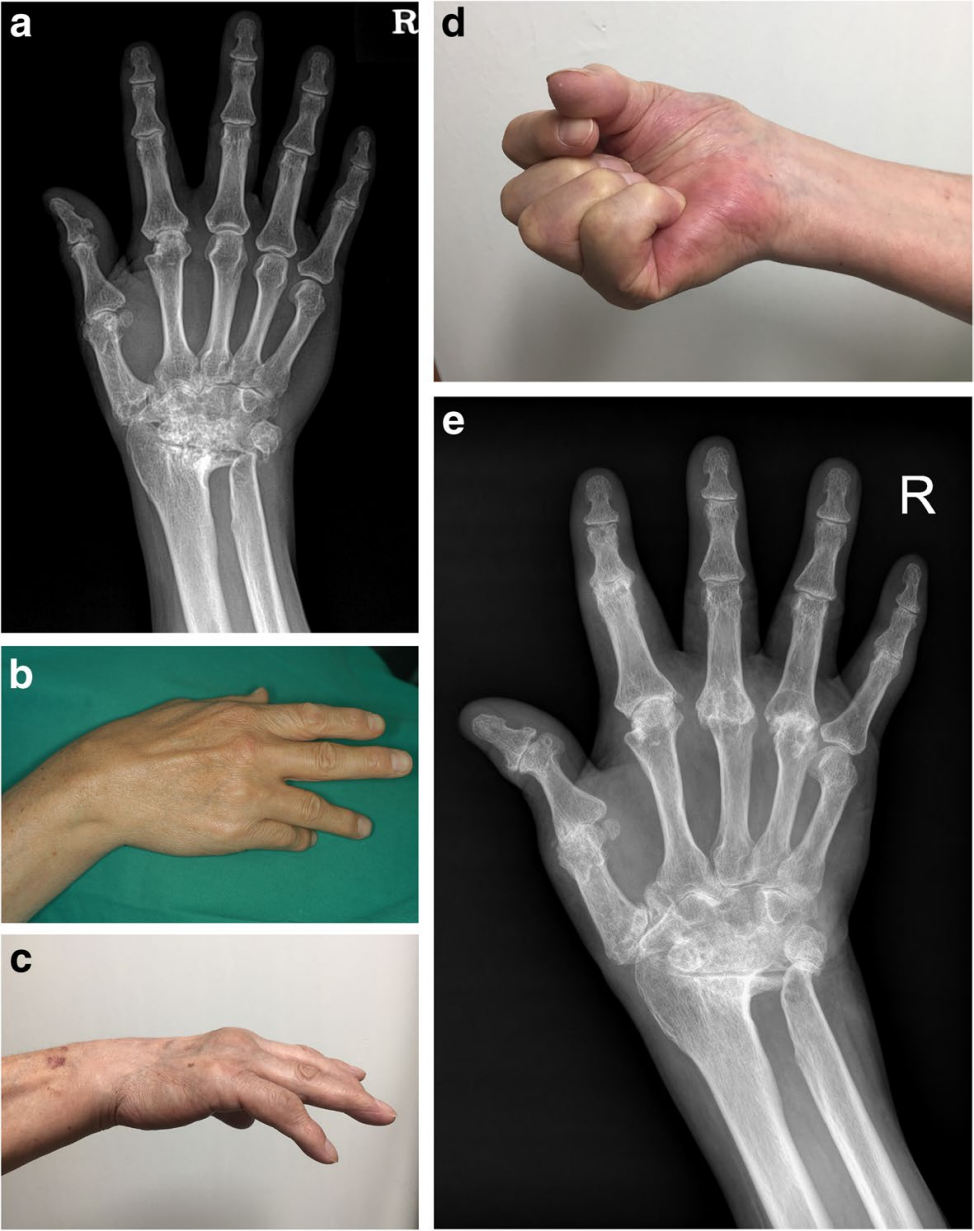
A 72-year-old male underwent tendon graft on the fourth and fifth fingers 141 months ago. **a** Preoperative wrist PA view. **b** Preoperative photograph showing extensor tendon ruptures on the fourth and fifth fingers. **c**, **d** Photographs at last visit show re-rupture on the extensor tendon of the fifth finger. **e** Wrist PA view taken at last outpatient visit

Excluding the two patients who had undergone additional surgery in a different hospital, two patients reported difficulties in daily activity (one due to deformity and another due to joint pain). The patient who chose not to undergo additional surgery had an extension lag on the fifth finger but reported no difficulties in daily activity (Fig. 2). Among the 13 patients who had switched jobs following the surgery, three patients reported issues with work: one due to deformity, one due to a re-rupture event, and one due to joint pain.

There were 17 cases of tendon transfer and 26 cases of tendon graft; there were no instances where both were used. There were no significant differences between the two groups with respect to age, duration of RA, time from rupture to surgery, and follow-up period. There were also no significant differences with respect to extension lag of the metacarpophalangeal joint, range of motion of the metacarpophalangeal joint, pulp-to-palm distance, Geldmacher score, and overall satisfaction rate (Table 2).

**Table 2:** Comparison of patient characteristics between the tendon transfer and tendon graft groups

Variable	Tendon transfer		Tendon graft		*P*-value
	Median	SD	Median	SD	
Age (years)	46.3	8.3	46.2	8.2	0.40
RA duration (years)	11.2	2.8	11.4	2.4	0.36
Time from rupture to surgery(weeks)	12.8	9.4	13.7	9.2	0.11
Follow-up (years)	16.0	5.1	13.9	3.3	0.10
Extension lag (°)	8.9	9.0	8.2	12.0	0.84
Range of motion (°)	71.1	17.9	77.3	17.0	0.12
Pulp-to-palm distance (cm)	0.3	0.4	0.4	0.7	0.51
Geldmacher score	20.9	3.9	20.7	3.7	0.48
Overall satisfaction rate	84.5	8.2	87.2	8.3	0.19

Time to surgery was significantly correlated with extension lag of the metacarpophalangeal joint (*R*^2^ = 0.437; *p* = 0.001) and pulp-to-palm distance(*R*^2^ = 0.156; *p* = 0.046) in the tendon graft group, but not in the tendon transfer group(*R*^2^ = 0.081; *p* = 0.269), (*R*^2^ = 0.033; *p* = 0.482). There was a significant correlation between extension lag of the metacarpophalangeal joint and overall satisfaction rate (*R*^2^ = 0.155; *p* = 0.009), but there was no relationship between pulp-to-palm distance and overall satisfaction rate(*R*^2^ = 0.074; *p* = 0.078) (Table 3).

**Table 3:** Simple linear regression analysis showing variables affecting clinical outcomes and overall satisfaction

Variable	*p*-value	Beta	R^2^
Tendon transfer group (ref. time from rupture to surgery)			
MP joint extension lag	0.269	0.352	0.082
Pulp-to palm distance	0.482	0.012	0.033
Tendon graft group (ref. time from rupture to surgery)			
MP joint extension lag	0.001	1.244	0.437
Pulp-to palm distance	0.046	0.047	0.156
Overall satisfaction			
MP joint extension lag	0.009	-0.029	0.155
Pulp-to palm distance	0.078	-0.337	0.074

## Discussion

Although both tendon transfer and graft are recognized as effective procedures, long-term follow-up results have not been reported. In this study, both tendon transfer and tendon graft for extensor tendon rupture in rheumatoid hands achieve favorable long-term outcomes.

Suzuki et al. compared between end-to-side and EIP transfer in patients with RA who had three extensor tendon ruptures and observed good results at 12 months postoperatively, with an average extension lag of 3° and an average metacarpophalangeal joint range of motion of 66° [12]. Chu et al. performed PL grafting in 14 patients and reported an improvement in extension lag from 38° preoperatively to 16° postoperatively in 54.1 months [13]. Satisfactory results were observed within a mean of 14 years of follow-up; the mean extension lag was 8.7°, mean pulp-to-palm distance, 0.4 cm; and mean overall satisfaction rate, 86.5.

The number of joint deformities is decreasing with advances in medical treatment [14], resulting in a reduced frequency of surgery in RA hands and wrists [15, 16]. Although this study does not account for the type of medication taken by patients, all patients were prescribed medication either at the authors’ hospital or elsewhere at the time of the final visit. Functional assessment could not be performed in only 3 of the 43 patients because of deformities of the metacarpophalangeal joint. Of the 87 digits, there were seven digits (3 cases) that re-ruptured, all of which occurred after a tendon graft. Given the need for two sutures in the proximal and distal segments, the risk of avascular necrosis of grafted tendons, as well as the use of existing inflammatory pathways in which tendon rupture has taken place, and the possibility of re-rupture may be higher in tendon graft procedures [17]. In addition, for each of the two re-rupture cases, there were three affected fingers.

Itubo et al. reported that the greater the number of affected extensor tendons, the greater the extension lag postoperatively [18]. Moreover, Sakuma et al. concluded that a higher number of ruptured tendons requires more complicated surgical procedures and rehabilitation [19]. Considering that the author used a method in which the ruptured distal tendons were sutured together and the PL was used as a bridge to connect the proximal tendon, it then follows that for a greater number of ruptured tendons, a larger strain on the implanted tendons or re-rupture may occur. However, the author of this study preferred tendon graft over tendon transfer, which resulted in twice the number of tendon grafts and a small number of re-ruptures. Therefore, further studies with a larger sample size are needed.

Two patients experienced difficulties in daily activities, and none with a reconstructed tendon reported an issue. The patient who chose not to undergo additional surgery following the re-rupture of the fifth digit reported no difficulties with everyday routines. Of the patients with joint deformity, only one had limited daily activities, which we believe is because the majority of RA patients adapt to hand deformities [20]. One patient had to resign from their job as an art teacher because of deformities in the metacarpophalangeal and interphalangeal joints, and those patients who had run grocery stores and restaurants could no longer work due to re-rupture and pain. All patients, aside from these three, maintained their previous jobs, not only because of positive progress postoperatively, but also because the majority of these patients were female and their jobs did not require strenuous use of the hand and wrist (e.g., manual labor). Although the surgical indications for RA are controversial, the need for surgery cannot be determined based on one factor alone. Therefore, a comprehensive, individualized approach that includes joint deformities, pain, daily activities, difficulties in the workplace, and the patient’s preference is needed.

Although tendon transfer is widely used to treat RA extensor tendon ruptures and is often the first-line option [5, 21], few studies have compared between tendon transfers and transfer grafts, and the results vary widely. We have previously reported no differences in the clinical results for tendon transfers and tendon grafts after an average follow-up period of 5.6 years [9]. In addition, Schaller et al. compared between 28 patients who underwent tendon transfer and 17 patients who underwent tendon graft. After 4.3 years of observation, they concluded that these techniques achieve comparable outcomes [17]. We also could not find significant differences between tendon transfers and tendon grafts after long-term follow-up. Meanwhile, Sakuma et al. reported negative clinical outcomes: longer surgery was delayed due to progressive contracture of the affected extensor muscles and flexion contracture of the affected joint [19]. In the present study, there was no significant correlation between time to surgery and clinical outcome (extension lag of the metacarpophalangeal joint and pulp-to-palm distance) in the tendon transfer group. However, a significant correlation was found in the tendon graft group. Considering that tendon transfer utilizes healthy, uninvolved muscles and tendons, the time to surgery is considered to be less important than the delay for a tendon graft. In three re-rupture cases, the time from rupture to surgery was on average 24 weeks and is considered a long time. Hence, it is important to evaluate the time to surgery when considering a tendon graft.

In this study, only the extension lag of the metacarpophalangeal joint showed a significant correlation with the overall satisfaction rate. Chung et al. found that the extension lag of the metacarpophalangeal joint was negatively correlated with patient satisfaction, whereas pulp-to-palm distance was not correlated [9]. Conversely, Nakamura et al. reported no correlation between extension lag of the metacarpophalangeal joint and patient satisfaction, whereas fingertip-to-palm distance was significantly correlated [6].

Schindele et al. demonstrated that flexion deficit is more problematic than minor extension lags; therefore, it is important to maintain finger flexion during the postoperative rehabilitation period [22]. However, Alderman et al. reported that the aesthetic aspects of the procedure are important in both male and female patients; thus, functional and aesthetic outcomes are also valuable [23]. The average age at the time of surgery for RA was 47 years; as such, most of the female patients in the current study had less physically strenuous activities of daily living or occupation compared with the general population. This could have contributed to the correlation between extension lag and subjective satisfaction, but not for pulp-to-palm distance.

This study had some limitations. First, this was a retrospective study. Second, this study did not reflect medication history or RA disease activity. Third, there were no criteria for performing tendon transfer or tendon grafting. Tendon transfer is one of the first options considered because of the low morbidity at the donor site, there is no need to consider the affected muscle function, and it requires only one suture site [21]. However, the author preferred tendon graft over transfer because the poor quality of the normal recipient tendon was often problematic, especially in the cases of end-to-side anastomoses. Further, tendons require a wide space to move if the distal stump of the ruptured tendon is short. In the case of tendon grafts, if performed shortly after rupture, the original muscle can be used to obtain the normal force prior to the operation. Finally, the rheumatology center of the hospital is familiar with tendon ruptures in RA, and thus, patients can undergo surgery within a relatively short amount of time. Prior studies have reported that tendon grafts are possible until 20 weeks after rupture [21], but in this study, the average time was 13 weeks. Further studies with more cases are needed to verify our results.

In conclusion, both tendon transfer and tendon graft for extensor tendon rupture in rheumatoid hands achieve favorable long-term outcomes, with no significant differences between the procedures. In cases of tendon graft, the time to surgery should be considered, and there is concern over re-rupture and extension lag of MP joint.

## Data Availability

The datasets during and/or analyzed during the current study are available from the corresponding author on reasonable request.
